# Proteome and transcriptome profile analysis reveals regulatory and stress-responsive networks in the russet fruit skin of sand pear

**DOI:** 10.1038/s41438-020-0242-3

**Published:** 2020-02-01

**Authors:** Yuezhi Wang, Meisong Dai, Danying Cai, Zebin Shi

**Affiliations:** Institute of Horticulture, Zhejiang Academy of Agricultural Sciences, Hangzhou, Zhejiang Province China

**Keywords:** Protein-protein interaction networks, Transcriptomics

## Abstract

The epidermal tissues of the cuticular membrane (CM) and periderm membrane (PM) confer first-line protection from environmental stresses in terrestrial plants. Although PM protection is essentially ubiquitous in plants, the protective mechanism, the function of many transcription factors and enzymes, and the genetic control of metabolic signaling pathways are poorly understood. Different microphenotypes and cellular components in russet (PM-covered) and green (CM-covered) fruit skins of pear were revealed by scanning and transmission electron microscopy. The two types of fruit skins showed distinct phytohormone accumulation, and different transcriptomic and proteomic profiles. The enriched pathways were detected by differentially expressed genes and proteins from the two omics analyses. A detailed analysis of the suberin biosynthesis pathways identified the regulatory signaling network, highlighting the general mechanisms required for periderm formation in russet fruit skin. The regulation of aquaporins at the protein level should play an important role in the specialized functions of russet fruit skin and PM-covered plant tissues.

## Introduction

When plants evolved from aquatic to terrestrial environments, they faced several challenges associated with survival, including water loss regulation, insulation against climatic variability, and protection against abiotic aggressions^1^. To adapt to environmental changes, specialized epidermal tissues, including the cuticular membrane (CM) and periderm membrane (PM), play a key role in terrestrial plants. The CM forms a continuous hydrophobic layer by waxes embedded in the cutin present on the outer face of the epidermal wall, conferring terrestrial adaptability in the primary organs (such as leaves, flowers, fruits, and primary stems)^2^. The PM is formed by stacked suberized cells that cover the secondary tissues as a protective layer. The suberized PM is known as “cork” in tree biology, and it is used worldwide in many industrial applications without proper substitutes for some of its properties^3^. In addition to the native periderm, a wound-healing periderm is commonly induced in injured tissues for insulation or protection from the environment. Differentiation of the peridermal layer of potato is a programmed senescence process of cell expansion, dramatic suberin deposition, and cell death^4^. The endoderm undergoes programmed cell death and abscission with the cortex, leading to the loosening of the outer tissues in the secondary growth of Arabidopsis roots^5^. The similar structure and common regulatory factors suggest that periderm development in different tissues and species may share homologous regulatory mechanisms. The PM is also sealed in fruits, such as pear, kiwifruit, and apple, and characterizes fruit quality by visual appearance and stress resistance. The different PM covers produce three types of pear fruit skin: russet, green, and intermediate. The primary skin of pear fruit is composed of three types of layers, a cork meristem, epidermal cells, and cuticle, with an inside out distribution. Bidirectional mutations between the traits of green and russet are found in pear fruit skin, making it a suitable material for resolving the regulatory mechanisms underlying PM and CM formation^6,7^.

Suberin is a lipid polymer that deposits inside the cell wall of the periderm as a critical barrier. Both suberin and cutin are polymers of fatty acid derivatives linked by ester bonds. However, suberin is characterized by the main components fatty alcohols, hydroxycinnamic acids (predominantly ferulate), and saturated aliphatics (chain length ≥ C20)^2^. Furthermore, suberized tissues also contain glycerol and insoluble hydrophobic residuals with high stability and limited depolymerization. The molecular mechanisms underlying russet formation were investigated by exploring the genes and pathways that are specific to the russet fruit skin of pear and apple^6,8–10^. The transcriptome of russet fruit skin was characterized by the upregulation of suberin deposition genes and stress-responsive genes, and the repression of cuticle biosynthesis genes compared with that of green fruit skin. A set of genes involved in different steps of the suberin biosynthesis pathway have been suggested to be key nodes, including transcription factor genes and enzyme-encoding genes involved in suberin biosynthesis, aromatic monomer inclusion, and substrate transport^10^. At least four MYB genes (MYB9, MYB107 (ref. ^10^), MYB93 (ref. ^11^), and MYB41 (ref. ^12^) are associated with the positive regulation of suberin biosynthesis. According to Lashbrooke et al.^10^, these regulatory mechanisms are homologous in angiosperms.

The accumulation of genomic data with high-throughput technologies has made it possible to apply genome-wide transcriptomic and proteomic analysis to interesting species. To date, transcriptome profiling in pear and apple fruit skin has been performed to understand russet development. Proteomic analysis can effectively address the limitations of transcriptome-based protein prediction by providing accurate quantitative and structural modification information about functional proteins and the dynamic state of the cell. However, a null proteomic study was carried out for pome skin russeting. Hence, in this study, an integrated tandem mass tag (TMT)-based proteomics and transcriptomics analysis was conducted to gain a precise global overview of pear fruit skin russeting and to identify the key nodes in regulatory networks that contribute to periderm formation and environmental adaptation.

## Materials and methods

### Plant material collection

**Fig. 1:**
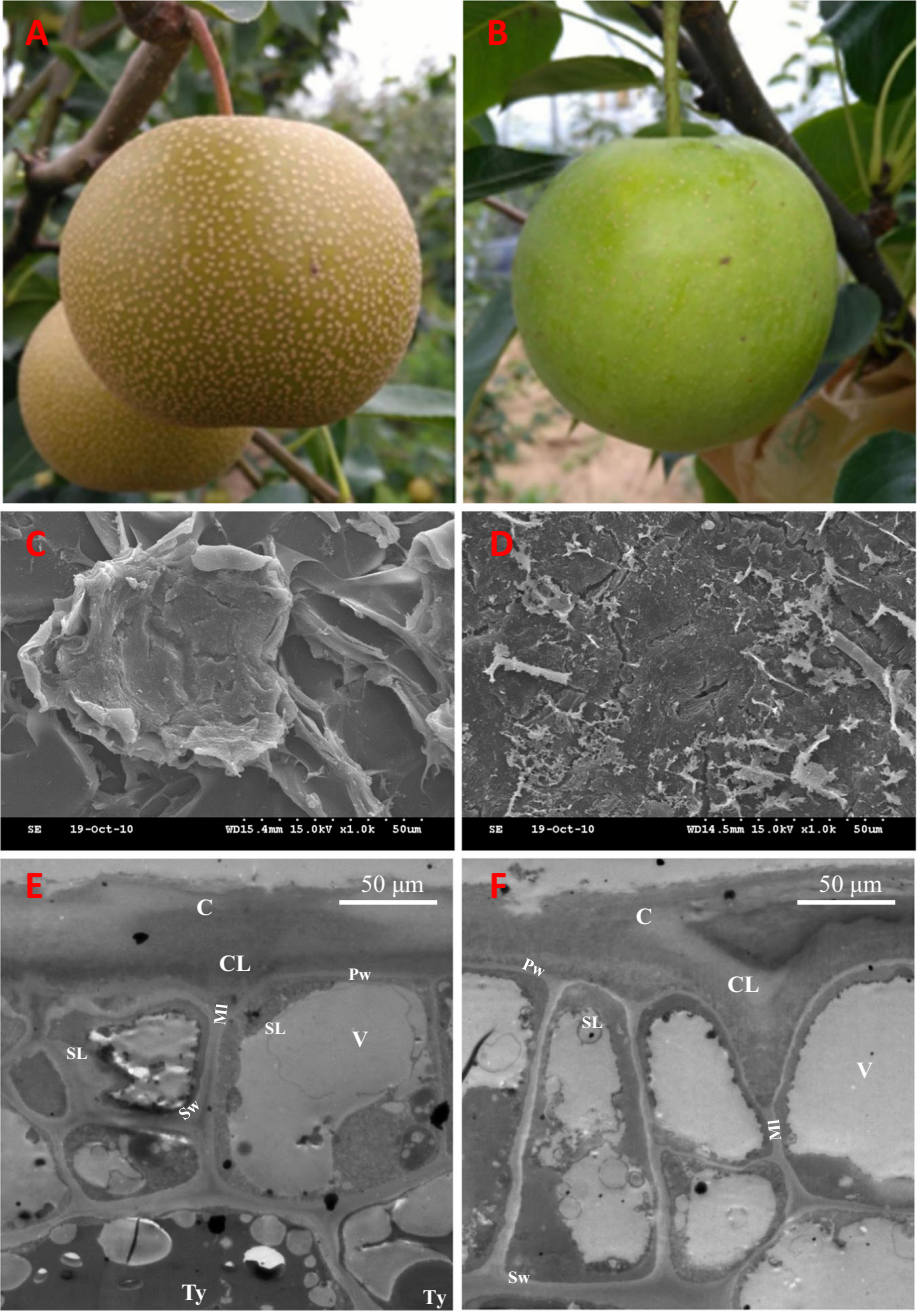
Phenotypes of the russet and green fruit skin of sand pear. The russet **a** and green **b** skin fruit of sand pear grown in an orchard. Scanning electron microscopy analysis of russet **c** and green **d** fruit surfaces showed that the skins of the two genotypes are covered by stacked suberized cells and cutin wax, respectively. Transmission electron microscopy analysis of russet **e** and green **f** fruit epidermal cells at the russet development stage showed that more tylosis (Ty) accumulated in the russet skin cells. C Cuticle; CL cuticular layer; Ml middle lamella; Pw primary wall; SL suberin layer; Sw secondary wall; V vacuole; Ty tylosis

Plant materials were prepared as described previously by Wang et al.^6^. Briefly, sand pear (*Pyrus pyrifolia*) fruit skin was obtained from 80-d russet fruits of ‘Niitaka’ pear (russet fruit skin; Fig. 1a) and its bud mutant line, ‘Suisho’ pear (green fruit skin) (Fig. 1b), growing in Yangdu Orchard (308290 N, 1208150 E, China) in June 2018. The same amount of skin from each fruit was collected and immediately stored in liquid nitrogen. For each sample, ten russet fruits or green fruits, and three biological replicates were separately used in phytohormone and molecular analysis. ‘Suisho’ pear has a similar phenotype and highly conserved genetic background to that of the wild-type ‘Niitaka’ pear, except for the fruit skin color mutation.

### Fruit postharvest water loss measurement

Ripe pear fruit stored at room conditions at 25 °C/18 °C (day/night) with 75% relative humidity were used in the experiment. The rate of water loss (RWL) was calculated using the formula RWL (%) = 100 × (FW_*t*1_−FW_*t*2_)/FW_*t*1_, where FW_*t*1_ = weight of the fruit at a certain storage time *t*1, and FW_*t*2_ = weight of the fruits at a certain storage time *t*2.

### Microscopy

Sample preparation for electron microscopy was performed according to the method described by Panikashvili et al.^13^. A scanning electron microscope KYKY-2800B SEM (KYKY Technology Co., Ltd., Beijing China) and a Hitachi H-7650 transmission electron microscope were used in the experiment. Calibrated micrographs were collected at two magnifications of 200× and 1000× with 20–25 KV acceleration potential. Transmission electron microscopy sections (70 nm) were observed.

### Hormone measurement

The contents of jasmonic acid (JA), salicylic acid (SA), and abscisic acid (ABA) were measured using the methods of You et al.^14^ at Zoonbio Biotechnology Co., Ltd, Nanjing, China. Briefly, ~1.5 g fruit skin samples were ground with 10 mL extraction buffer in a cooled mortar and then shaken for 30 min at 4 °C. After adding 20 mL dichloromethane, the mixture was shaken again. Then, the sample was centrifuged at 4 °C and 13,000 rpm for 5 min, and the lower organic phase was kept and dried with liquid nitrogen in the dark. A volume of 400 μL methanol (0.1% methane acid) was used to dissolve the dried pellets. Then, the product was detected by combined high-performance liquid chromatography-mass spectrometry/mass spectrometry (HPLC-MS/MS) after 0.22 micron filtration. HPLC-MS/MS analysis was carried out for the purified product with a 2 μL injection volume. The MS conditions were as follows: spray voltage, 4,500 V; air pressure, 15 psi; nebulizer pressure, 65 psi; pressure of aux, 70 psi; and atomizing temperature, 400 °C.

### Complementary DNA library construction and sequencing

Approximately 5 g of each fruit skin was frozen and ground in nitrogen, and the total RNA was extracted according to the manufacturer’s illustration of the plant RNA kit (Invitrogen, CA, USA). Each library was prepared using 1 µg RNA per sample according to the recommendations of the NEB Next® Ultra™ RNA Library Prep Kit (NEB, USA). Library sequences were indexed, and sequencing was carried out on a 150 paired-end Illumina platform.

### Sequence and expression analysis

All RNA-Seq data were deposited in the National Center for Biotechnology Information (NCBI) database under the accession GSE139895. Data quality control was performed by removing adapters and discarding poor quality reads with quality insert fragment <20 bp or the proportion of *N* > 10% from the raw data. The alignment of the selected reads with the reference genome of Chinese white pear (*Pyrus* × *bretschneideri*) deposited in the NCBI database was carried out by Hisat2 v2.0.4 (ref. ^15^). Transcripts were assembled and annotated from the read alignment results by Cufflinks v2.1.1 (ref. ^16^) with the method based on the reference annotation. The gene expression levels were quantified with the fragments per kilobase of exon per million fragments mapped method^8^. The DESeq^17^ R package (1.18.0)^18^ was employed for differential gene expression analysis, and Benjamini and Hochberg’s false discovery rate was used for multiple test correction. Only the genes with corrected *P* < 0.05 were considered to be differentially expressed.

### Peptide preparation

Each sample was individually milled into powder in a mortar with liquid nitrogen. Protein extraction was carried out according to the published method^19^, followed by trypsin digestion and TMT reagent labeling. Fractionation of the tryptic peptides was conducted with the Agilent 300 Extend C18 column and a high pH reversed-phase HPLC system.

### LC-MS/MS analysis and database searching

The peptides were first analyzed by Q-exactive HF-X MS (Thermo Fisher Scientific) coupled with a Rigol L3000 HPLC system (Rigol, Inc., Beijing, China). Then, the obtained peptide sequences were searched against the reference genome of Chinese white pear (*Pyrus* × *bretschneideri*; 47,086 sequences) deposited in the NCBI database.

### Functional analysis of differentially expressed proteins and genes

The public nonredundant database of proteins (including Pfam, ProDom, SMART, PRINTS, PANTHER, and ProSiteProfiles) was used for the functional annotation of all selected proteins with InterPro and Gene Ontology (GO) analysis^20^. Protein family analysis was conducted using the Clusters of Orthologous Groups database, and pathway analysis was performed with the KEGG database (Kyoto Encyclopedia of Genes and Genomes). GO enrichment was analyzed by the GOseq^21^ R package with gene length bias corrected. The corrected *P*_0.05_ was selected as a significant criterion for all GO terms enriched by differentially expressed genes (DEGs). KEGG enrichment of DEGs was determined using KOBAS^22^ software. The online server STRING-db (http://string.embl.de/) was used to predict the probable interacting partners with reference species. All enrichment analyses mentioned above were conducted by the enrichment pipeline^23^.

## Results and analysis

### Phenotype characteristics of the russet and green fruit skin of sand pear

The color of all pear fruit skins was green during the early stages, i.e., in the young fruit, but part of the genotypes were differentiated into russet or intermediate types during subsequent development. Observation with scanning electron microscopy revealed a thick waxy layer on the skin of the green fruit of sand pear, but the russet skin consisted of layers of dead cells, with the outer layer presenting as a loose lamellar structure (Fig. 1c, d). Transmission electron microscopy analysis revealed a putative suberin layer (SL) in the epidermal cells of both types of fruit skins, but more tylosis (Ty) accumulated in the epidermal cells of russet skin than in those of green skin (Fig. 1e, f).

**Fig. 2:**
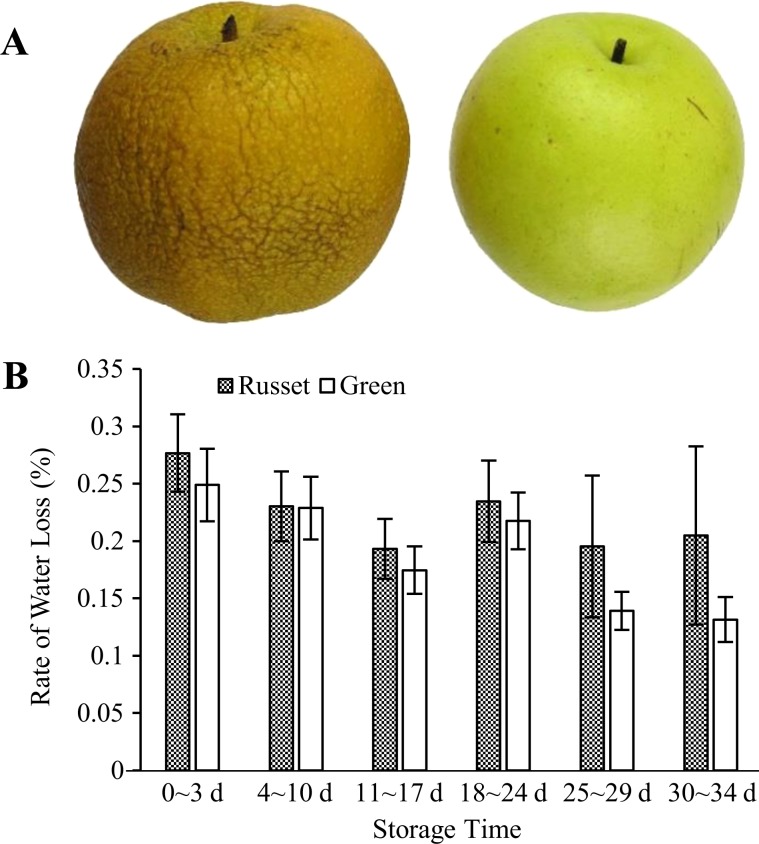
RWL of the russet and green skin fruit of sand pear at different storage periods under room conditions after harvest. After storage under room conditions, the russet fruit skin exhibited obvious shrinkage due to water loss **a**. The reported value is the mean ± SE **b**

In general, pear fruit with green skin has higher storage resistance than does russet skin fruit. After a period of storage, the russet fruit skin of sand pear appeared to shrink until the occurrence of putrefaction (Fig. 2a). Under room conditions, the RWL of russet skin fruit was higher than that of green skin fruit. The longer the storage time was, the greater the difference in RWL between the two types of pear fruit (Fig. 2b). The RWL of both russet skin and green skin fruit showed a decreasing trend during storage, but it decreased more in green skin fruit than in russet skin fruit from 25–29 d, which resulted in a larger RWL difference between the two types of fruit. Green skin fruit had a better ability to maintain water during storage.

### Differential accumulation of JA, SA, and ABA between russet and green fruit skin

**Fig. 3:**
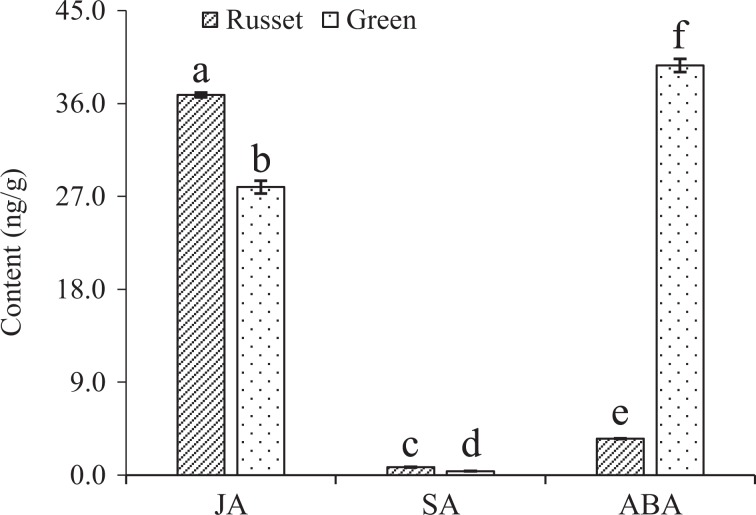
Differential accumulations of JA, SA, and ABA between the russet and green fruit skin of sand pear. Different significant differences from the control at *P*_0.05_ (Tukey’s test). The error bar is too small to be seen for c, d, and e

To analyze the signal characteristics of endogenous hormones between russet and green fruit skin, we compared their accumulation of the hormones JA, SA, and ABA that are involved in plant biotic and abiotic stress responses. The results showed significant differences in the three hormones between russet and green fruit skin. JA and SA accumulated more, while ABA was dramatically decreased in russet fruit skin compared with green fruit skin (Fig. 3).

### Transcriptome and proteome profiles of the skin of pear fruit

The RNA-Seq generated 51.97 Gigabyte (GB) of clean data (Q30 > 93.66%) with 7.2–9.8 GB from the six complementary DNA (cDNA) libraries. A total of 47,991,052–65,340,486 quality reads per library were obtained, and 68.17–68.68% were unique loci in the reference genome. Among all 42967 gene loci in the pear reference genome detected by read alignment (had at least one read) in the libraries of pear fruit skin, 772 new gene loci were initially identified in this study (Supplementary Table 1). The high Pearson’s correlation coefficients (PCCs; Supplementary Fig. 1) indicate the quality control.

Protein profile analysis produced 72,037 peptide spectra from the fruit skin libraries, resulting in 11,174 proteins and the quantification of 11,157 proteins. High PCCs with *R*^2^ from 0.983 to 0.997 among the three biological replicates indicated that the expression patterns of the replicates shared a high degree of similarity, and the experiment was carried out under good control. Correlation analysis was also performed at the transcriptome and proteome levels by comparing the fold changes in expression between the two types of fruit skins. Correlations were revealed between 10,881 pairs of mRNAs and their coding proteins (*γ* = 0.588, *R*^2^ = 0.253, Supplementary Fig. 2).

The transcript analysis of the two genotypes by the DESeq R package identified 3885 DEGs (above 2-fold changes [*P* < 0.05]), including 2020 induced and 1865 repressed DEGs in russet skin (Supplementary Table 1, Supplementary Fig. 3l). Additionally, 275 differentially expressed proteins with fold changes higher than two (*P* < 0.05) were identified, 124 of which were induced and 151 of which were repressed in russet skin (Supplementary Fig. 3r).

### DEG GO enrichment between the russet and green fruit skin of sand pear

The DEGs revealed by the transcriptome analysis were categorized by GO enrichment analysis with the GOseq method^21^. Only seven biological process classes and ten molecular function classes were selected for the next round of analysis by more stringent screening criteria at the corrected *P*-value cutoff of 0.05 (Supplementary Table 2). The selected GO classes included lipid biosynthesis and metabolic processes and oxidation–reduction process. Similarly, at the protein level, six biological process classes, one cellular component class, and five molecular function classes were selected for the next round of analysis by the corrected *P*-value cutoff of 0.05 (Supplementary File 3). The GO classes (GO:0006629, GO:0006633, GO:0008610, and GO:0044255) of lipid biosynthesis and metabolic processes were commonly enriched at both the transcript and protein levels. Moreover, different enrichment of GO classes was also detected at the transcript and protein levels, such as the transcript-specific enrichment of the GO class oxidation–reduction process (GO:0055114) and the protein-specific enrichment of the GO classcarboxylic acid biosynthetic process (GO:0046394). For proteins, other enriched GO classes with a *P*-value cutoff of 0.05 included defense response, response to water, transport, etc. These results provided a multidimensional profile of metabolic and regulation characteristics at both the transcript and protein levels for sand pear fruit skin.

### Comparison of the genes involved in the enriched biological processes between the russet and green fruit skin at transcript and protein levels

**Fig. 4:**
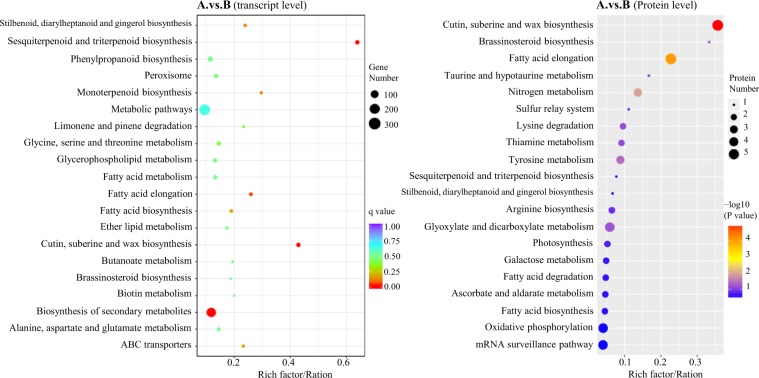
Bubble diagrams of the KEGG enrichment of DEGs between russet (A) and green (B) fruit skin at the transcript and protein levels. The abscissa is the ratio of the number of DEGs in the pathway to that of total genes identified. A larger value indicates the higher concentration of DEGs in the pathway. The color shade of the point represents the change in the *P*-value determined by a hypergeometric test. A smaller *P*-value indicates more statistical significance and greater test reliability. The dot size indicates the number of DEGs in certain pathways. A larger size represents more DEGs

Previous studies that investigated transcripts demonstrated that the differentiation between russet and green fruit skin in sand pear is controlled by a relatively complex network that mainly regulated diverse genes involved in cutin, suberine and wax biosynthesis, fatty acid elongation, ABC transporters, sesquiterpenoid and triterpenoid biosynthesis, and so on^6,7^. However, in this study, the KEGG enrichment analysis at the transcript level showed a high consistency with the results of the previous transcriptome analysis, but some pathways were not enriched at the protein level (Fig. 4, Supplementary Tables 4 and 5). In these interesting pathways, the number of DEGs at the protein level was significantly smaller than that at the transcription level, and most of the genes were differentially expression in the transcriptome analysis, but showed no significant difference at the protein level between the two types of fruit skins.

#### Regulation of the expression of genes involved in cutin, suberine, and wax biosynthesis between the two types of fruit skins

**Fig. 5:**
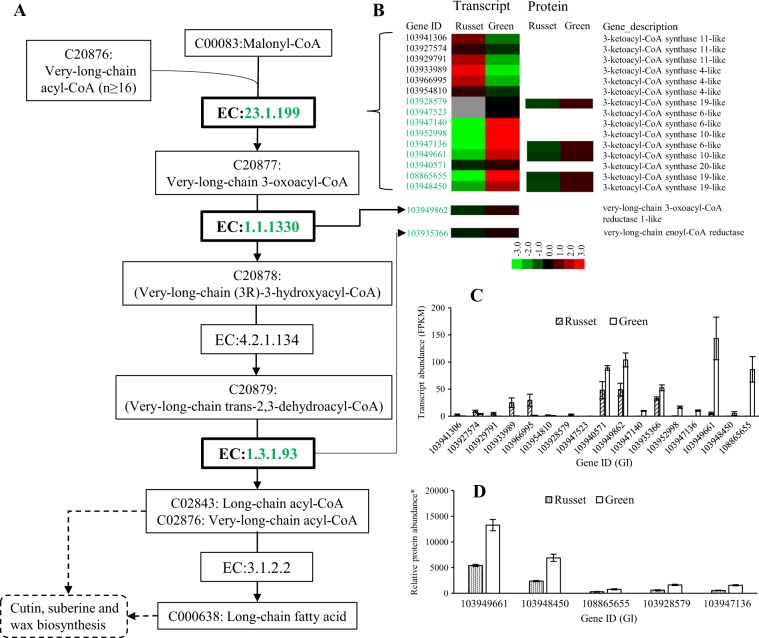
Schematic of the transcriptome and proteome revealing the regulation of the long fatty acid biosynthesis pathway in the russet fruit skin of sand pear. **a** A detailed diagram of long fatty acid biosynthesis including the subset of nodes or metabolites and enzymes that are involved in the process. The enzymes with repressed expression in russet fruit skin at the transcript and/or protein levels are shown in green. **b** Heat map of differentially expressed transcripts or proteins. Transcript **c** and protein abundance **d** of russet-related genes involved in long fatty acid biosynthesis

**Fig. 6:**
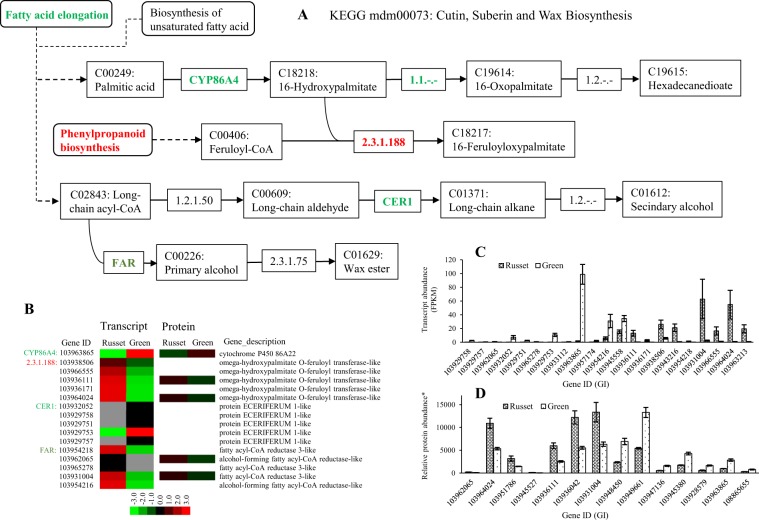
Schematic of the transcriptome and proteome revealed the regulation of the pathway map00073 for cutin, suberin, and wax biosynthesis in russet fruit skin in sand pear. **a** A detailed diagram of cutin, suberin, and wax biosynthesis, including the subset of nodes or metabolites and enzymes involved in the process. Enzymes with repressed or enhanced expression in russet fruit skin at the transcript and/or protein levels are shown in green or red, respectively. **b** Heat map of the differentially expressed transcripts or proteins. Transcript **c** and protein abundance **d** of russet-related genes involved in cutin, suberin, and wax biosynthesis

**Fig. 7:**
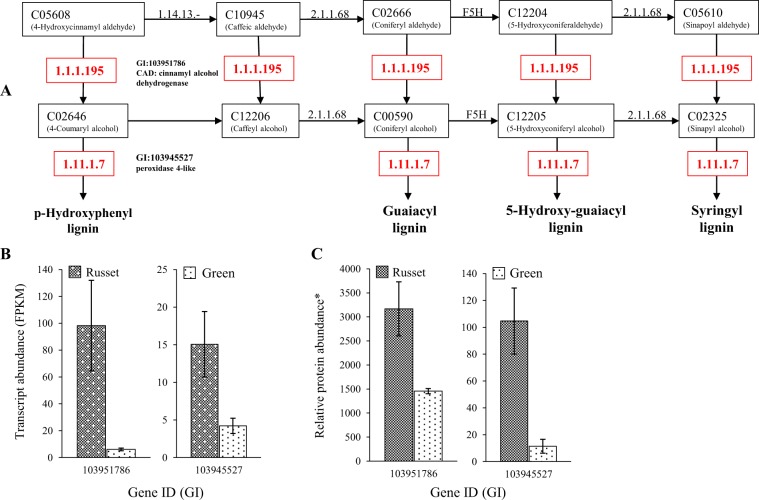
Regulation of the phenylpropanoid biosynthesis pathway in the russet fruit skin of sand pear. **a** A partial diagram of phenylpropanoid biosynthesis constructed based on the KEGG pathway and gene expression data. Enzymes with enhanced expression in the russet fruit skin at the transcript and protein levels are shown in red. Transcript **b** and protein abundance **c** of russet-related genes involved in phenylpropanoid biosynthesis

Both fatty acid elongation and cutin, suberine, and wax biosynthesis, which are involved in PM and CM formation, were enriched in the protein and transcript analysis. The regulated genes in the pathways represented the key nodes and regulatory profiles for epidermal tissue formation in pear fruit skin. The fatty acid elongation pathway is upstream of cutin, suberine, and wax biosynthesis, as it supplies the substrate for the biosynthesis of the long fatty acids cutin, suberine, and wax. The upstream enzyme EC: 23.1.199 (3-ketoacyl-coa synthase, KCS) catalyzes the key step of long fatty acid biosynthesis (Fig. 5). A total of 15 genes encoding KCS showed low expression at the transcript level; the expression of 9 genes was repressed, but the expression of 6 genes was enhanced in russet fruit skin. The five putative KCS genes detected were all repressed at the protein level in russet fruit skin (Fig. 5b, c). Both the KCS 10-like gene (103949661) and the KCS 19-like gene (103948450) were highly expressed at the protein level in green fruit skin (Fig. 5d). The other two enzymes (EC: 1.1.1330 and EC: 1.3.1.93) in the downstream reaction were also repressed at the transcription level in russet skin, but no protein expression was detected in either tissue type. In addition to being limited by the repression of the upstream pathway, map00073 was also regulated by additional enzymes involved in biosynthesis (Fig. 6). The upstream enzyme CYP86A (GI: 103963865) showed repressed expression at the transcript and protein levels in russet fruit skin compared with green fruit skin. Similar regulation was also observed in the expression of five ECERIFERUM one-like genes. However, two genes (103931004 and 103962065) encoding enzymes involved in wax ester (C01609) biosynthesis showed more transcript and protein abundance in russet skin. All five genes encoding putative omega-hydroxypalmitate O-feruloyl transferase were induced at both the protein and/or transcript level in russet fruit skin. The outcome of map00940 was considered another source of C18217 (16-feruloyloxypalmitate)-catalyzed by omega-hydroxypalmitate O-feruloyl transferase (Fig. 7). The enzymes EC: 1.1.1.195 and EC: 1.11.1.7 catalyzed reactions involved in lignin biosynthesis, which was in line with the upregulation in russet fruit skin of EC: 2.3.1.188 (Fig. 6). Gly-Asp-Ser-Leu (GDSL) lipases are required for lipid catabolic processes in the fruit cuticle and for plant defense responses, in which lipids have a potential role as signaling molecules^24–27^. Expression studies identified 28 GDSL-type lipase genes with varied expression at the transcript and protein levels between the two types of fruit skin (Supplementary Table 6). Phylogenetic analysis showed the clustering of GDSL-type lipase into nine types, as reported in Arabidopsis^28^, and a new type of Va that consisted of two members was identified (Supplementary Table 6, Supplementary Fig. 4). Among the DEGs, genes in type I, II, and III were generally downregulated in russet fruit skin at the protein level, but the two IIc-type genes showed more transcript and protein abundance in russet fruit skin.

#### Differential transcript expression but conserved protein expression of ABC transporter genes in the two types of fruit skins

ABC transporters play essential roles in protecting layer formation and in transporting phytohormones^29^. Differential expression of ABC transporter genes at the transcript level was observed between the russet and green fruit skin of sand pear^6,8^. In this study, the expression of all eight types of ABC transporters, including a total of 65 members, was at the transcript and protein levels. Among them, 18 genes were differentially expressed at the transcript level, and only *ABCA* (GI: 103938290) was differentially expressed at the protein level between the two types of fruit skin (Supplementary Table 7). In addition, 87 ABC transporter genes were detected at the transcript level but not at the protein level. All the genes without detectable protein expression were also expressed at low levels at the transcript level.

#### Stress-responsive genes and signaling in russeting skin of sand pear fruit

**Fig. 8:**
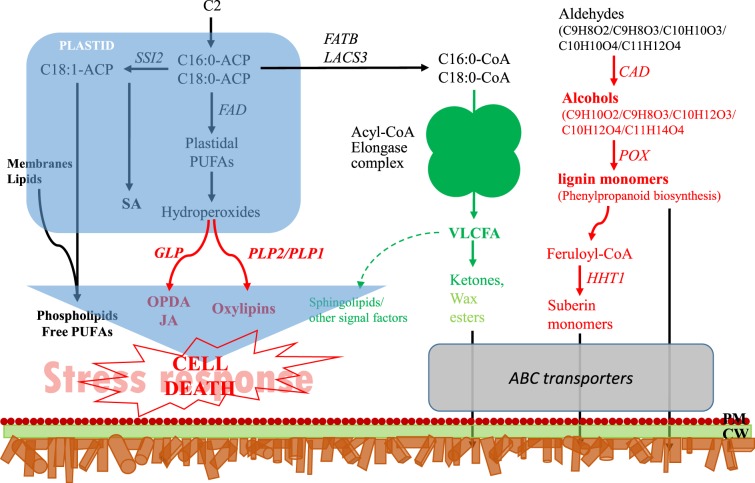
Schematic diagram summarizing the metabolism and signaling in the russet fruit skin of sand pear. The diagram was constructed based on the metabolic pathways summarized in the hypersensitive cell death response in Arabidopsis by Raffaele et al.^65^. Enzymes and partial diagrams marked in red or green indicate strongly enhanced or repressed regulation, respectively, in russet fruit skin at the transcript and/or protein levels. The differential regulation of wax esters in russet skin are indicated in red. We hypothesize that a low content of VLCFAs represses the biosynthesis of signaling molecules that could block or alter the signaling pathway, as indicated by the dotted arrow. PM plasma membrane; CW cell wall

**Fig. 9:**
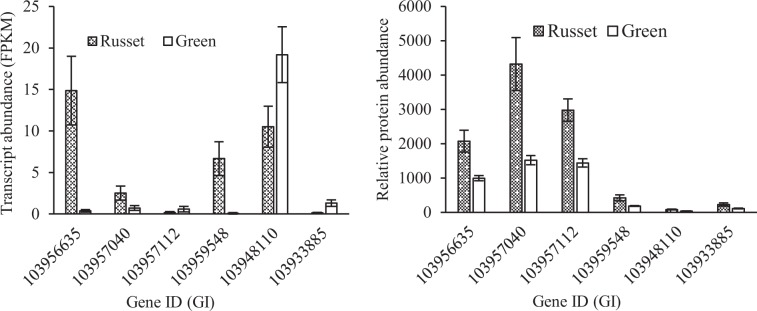
Enhanced expression of PLP2 genes (Gene ID: 103956635, 103957040, 103957112, and 103959548) and CRK genes (GI: 694350157 and 694428606) at the transcript and/or protein levels in russet fruit skin of sand pear

The metabolic characteristics of the russet fruit skin of sand pear are summarized in Fig. 8. The above-mentioned results indicated that the differences in the molecular metabolic pathways between the russet and green fruit skin of sand pear caused differences in substance accumulation, which subsequently caused differences in cellular differentiation. Patatin-like protein 2 (PLP2) was proven to be necessary for spontaneous cell death in plants by producing the precursors of fatty acids for the specific oxylipins that led to distinct cellular functions and consequent pathogen resistance^30^. In the russet fruit skin of sand pear, the expression of four putative *PLP* genes (Gene ID: 103956635, 103957040, 103957112, and 103959548) was significantly upregulated at both the transcript and protein levels (Fig. 9), which was consistent with the role of programmed cell death in the russet skin. Therefore, cell death in russet skin is induced by the absence of adequate protection by the CM. The dying cells are considered to produce secondary signals that strongly induce cooperative defense responses in surrounding surviving cells^31,32^. The suberization of epidermal cells provides physical and biochemical barriers and secondary signals by its own death to protect neighboring living tissues. Cystine-rich protein kinases (CRKs) are synthesized upon pathogen perception, mediating the BAK1-dependent cell death associated with the FLS2 complex, and these proteins act in coordination to enhance plant immune responses^33^. Two *CRK*s were detected with more abundant transcripts in the green fruit skin, but higher protein levels in russet fruit skin (Fig. 9).

**Fig. 10:**
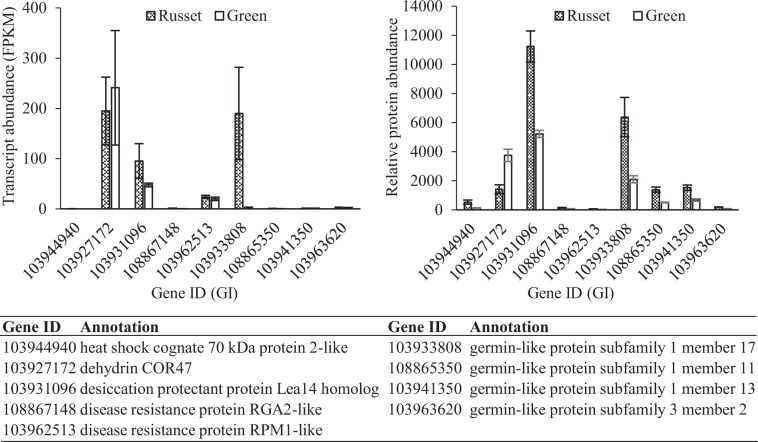
Expression characteristics of stress-responsive genes at the transcript and protein levels in the fruit skin of sand pear. Except for 103927172, all these genes were strongly induced at the protein level in russet fruit skin

**Fig. 11:**
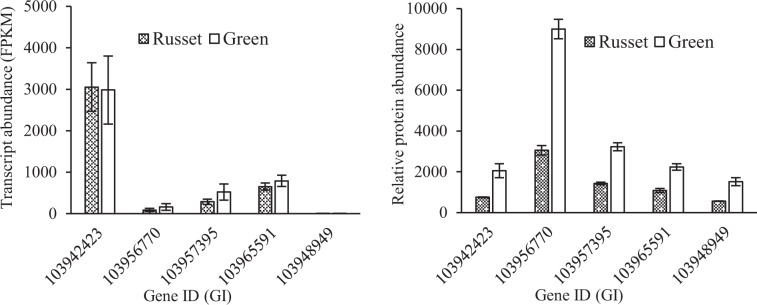
Differential regulation of aquaporin genes at the transcript and protein levels in the russet and green fruit skin of sand pear. All these genes showed constant transcript levels but were strongly repressed at the protein level in russet fruit skin compared with green fruit skin of sand pear

The germin-like protein (*GLP*) gene family is an important defense gene family associated with the JA-dependent pathway^34^. In this study, the expression of four *GLP* genes (Gene ID: 103933808, 108865350, 103941350, and 103963620) was significantly enhanced at both the transcript and protein levels, and the expression of 103933808 was obviously higher than that of the other *GLP* genes (Fig. 10). Triterpenoids have a potential role in the protection against pathogens and pests due to their antimicrobial activity^35–37^. Triterpene caffeates with anti-inflammatory activities have been identified in pear and apple with preferential expression in russet skin^36^. The transcriptome and proteome comparison of the two types of fruit skin showed the enrichment of sesquiterpenoid and triterpenoid biosynthesis. Among a total of 23 genes with differential expression in the pathway, all 19 beta-amyrin synthase-like genes, except 103967646 and 103927760, were upregulated in russet skin at the transcript level. The differential expression of these beta-amyrin synthase-like genes was also detected at the protein level (Supplementary Table 8). Responses to abiotic stress tolerance genes were also detected in the russet fruit skin of sand pear. The expression of aquaporin genes was repressed, while that of the LEA gene was enhanced at the transcript and/or protein levels (Figs. 10 and 11). All ABA-responsive genes showed no enrichment at either the transcript or protein level, which was in accordance with the low ABA level in russet fruit skin. Instead, the gene (GI: 103960819) encoding putative cytochrome P450 90B1 was involved in brassinosteroid biosynthesis and was more highly expressed at the transcript and protein level in the russet fruit skin of sand pear (Supplementary Tables 4 and 5).

## Discussion

The protective barrier provided by the periderm in most secondary tissues confers a key environmental stress adaptability for eudicots and gymnosperms. In this study, the molecular mechanisms underlying PM formation were explored by proteome and transcriptome analysis and by comparing the wild-type PM-covered (russet) fruit skin and the mutant CM-covered (green) fruit skin in sand pear. The analysis of gene expression correlation and GO and KEGG enrichment at the transcript level showed high consistency with the results of previous transcriptome studies (Fig. 2, Supplementary Fig. 5). However, a relatively low correlation was detected between the expression regulation at the transcriptome and proteome levels in the two types of fruit skins (Supplementary Fig. 2). Specific regulation at the protein level should play a fundamental role in the biological function of the PM or CM of pear fruit skin. The combined omics analysis effectively identified interesting genes and pathways and identified new functional genes that regulate protein levels in the russet fruit skin of sand pear.

### Metabolic characteristics and abiotic stress-responsive genes in russet skin

Similar to that the differentiation of potato periderm, the differentiation of the russet fruit skin of sand pear involves cell expansion, intense deposition of suberin, and a programmed cell death. The differences in the chemical components in russet and green fruit skin was analyzed via gas chromatography–MS in apple^10^. The results revealed a significant decrease in the abundance of C16-v-HFA and C16–9/10,16-DHFA monomers but a high content of C20- and C22-v-HFA in russet fruit skin. The content of phenolics and organic acids (including cinnamic acid, ferulic acid, and benzoic acid) showed a consistent increase with increasing levels of longer chain length substances in russet fruit skin. These results indicated the higher accumulation of suberin in the russet fruit skin. The high suberin content of the cell wall is regarded to be the most relevant characteristic of the periderm in its chemical composition^38,39^. In addition, secondary cell wall lignification thickening and Ty accumulation were also found in the cells of russet fruit skin (Fig. 1). Our previous study revealed higher contents of lignin, cellulose, and hemicellulose but lower levels of pectin in russet fruit skin than in green fruit skin with Fourier transform infrared spectroscopy analysis^6^.

The omics analysis of the russet fruit skin of sand pear revealed the enhanced biosynthesis of suberin but the repressed biosynthesis of cutin (Fig. 6). The repressed biosynthesis of cutin was controlled by a signaling cascade (Figs. 4 and 5). First, in the upstream pathway, the repressed expression of the Elongase complex (EC: 23.1.199, EC: 1.1.1330, and EC: 1.3.1.93) inhibited the biosynthesis of long chain fatty acids, providing substrates for the biosynthesis of cutin, wax, and suberin. CER1 is a core component of the complex controlling very-long-chain alkane synthesis^40–42^. CYP86A4 plays a key role as a cutin ω-hydroxylase in the biosynthesis of 16-hydroxypalmitate, 10,16-dihydroxypalmitate, and 1,16-hexadecanedioic acid^43^. Therefore, the downregulation of CER1 and CYP86A4 homolog genes decreased the production of cutin monomers. It was unexpected that the expression of putative CER1 proteins was not detected in either russet or green fruit skins. The (poly)phenolic and (poly)aliphatic domains of suberin showed specific chemical composition and distinct tissue and subcellular location^44^. The products from the phenylpropanoid biosynthesis pathway supply the substrate for the (poly)aliphatic domain in suberin biosynthesis, which was upregulated at both the transcript and protein levels (Fig. 7). This in turn caused the increased accumulation of lignin and putative (poly)aliphatic domain suberin in the russet fruit skin of sand pear.

Different from the physical and chemical properties of the CM, the PM that covers russet fruit skin has a loose lamellar structure of stacked cork cells with high plasticity and water permeability^45,46^ (Figs. 1c and 2b), which should be the main factor involved in the RWL difference between the two types of fruit. Despite its importance, the underlying mechanisms of PM stress defense and regulation are largely unknown. In this study, certain genes and metabolic pathways were detected by combined omics analysis, revealing a framework of mechanisms for the environmental adaptation of russet fruit skin. Without CM protection, russet skin fruit had a higher RWL than did green skin fruit (Fig. 2), subjecting russet skin fruit to dehydration stress. As a response, the expression of aquaporins in both the plasma membrane and tonoplasts was repressed at the protein level (Fig. 11), hindering water loss through epidermal cells and reestablishing the water balance of russet skin fruit. The downregulation of aquaporins is generally considered to reduce cell permeability with low water loss to maintain plant water requirements in a dry environment^47^. The enhanced expression of desiccation protectant protein Lea14 homolog (GI: 103931096) and heat shock cognate 70 kDa protein two-like (GI: 103944940; Fig. 10) could also protect russet skin fruit from abiotic stresses. ABA, as an abiotic stress signal, regulates the plant molecular response to water loss. It was unexpected that the ABA concentration in russet fruit skin was obviously lower than that in green fruit skin (Fig. 3), which suggested an ABA-independent pathway for stress response gene regulation in russet fruit skin. The different ABA signals may be a response of the larger RWL difference between the two types of fruit from 25–29 d of storage.

### Putative protective barriers to biotic stresses in russet skin

The russet skin of sand pear fruit possesses at least three types of protective barriers to biotic stresses, which include the physical barrier formed by the periderm and the chemical barrier formed by the accumulation of certain metabolites, such as triterpenoids that have antimicrobial activities. The GLP gene has been proven to be one of the important defense genes involved in plant basal host resistance^34,48–50^. In this study, the expression of GLP genes was significantly upregulated in the russet fruit skin of sand pear, conferring higher basal host resistance in the organ. GLP plays a role in the production of H_2_O_2_, which in turn acts as a signaling molecule at the infection site to induce a range of defense responses^50–54^. The overexpression of *OsGLP2–1* increased the accumulation of endogenous JA and induced the expression of defense-related genes in the JA-dependent signaling pathway^34^. *RPM1* acts as a disease resistance (R) gene that confers plant pathogen recognition and resistance^55^. An RPM1-dependent local stimulus can generate a rapid signal flow through systemically responding tissues to mediate a rapid response via transcription and de novo JA biosynthesis^56^. Therefore, the higher expression level of the RPM1-like gene (GI: 103962513) suggested an upstream stress response and enhanced JA biosynthesis in the russet fruit skin of sand pear. Consistent with this speculation, the expression of the RGA2-like gene (GI: 108867148), which is potentially controlled by the JA-dependent signaling pathway, was also upregulated in russet fruit skin.

### Functions of ABC transporters in the development of sand pear fruit skin

The transcript and/or protein expression of ABC transporters suggests their putative roles in pear fruit skin formation. Homologs of the ABC transporters involved in the transport of cutin, suberin, and lignin monomers were detected in both previous studies^6,8^ and in this study. Homologs with simultaneous expression may be functionally redundant in pear fruit skin. Differences in ABA, JA, and SA levels were revealed between the russet and green fruit skin. Certain ABC transporters have been proven to be involved in phytohormone transportation^29^, which could play a role in hormone regulation in pear fruit skin. However, the expression of the homologs of the identified ABC transporters for ABA or JA was low or undetectable in both types of fruit skins. Due to the large number and different types of ABC transporters expressed in fruit skin, except for functional redundancy, the functional differentiation and cooperation between the different members need to be revealed in further studies.

### Transcription factors involved in trait formation

Although the russeting of fruit skin involves the metabolic control of certain biochemical substances and the programmed cell death of the epidermis, it is still considered a quality trait that is genetically controlled by a single gene; in contrast, the semirusset trait is a quantitative trait that is controlled by multiple loci^7^. Different MYB transcription factors were revealed to be regulators of either suberin or extracellular lipid biosynthesis and accumulation in plants. MYB41 is a master regulator of the specific synthesis and assembly of suberin during certain tissue and cell differentiation^12^. MYB107 positively regulated suberin biosynthesis in the seed coat^10,57^. MYB93 regulated the development of lateral roots, exocarp russeting, and ectopically induced suberization^11,58^. The upregulation of MYB1 in cork tissues showed its association with secondary growth and, in particular, with the cork biosynthesis process^59^. Our datasets identified 12 MYB-coding genes homologous to the mentioned MYB regulators involved in plant tissue suberization (Supplemental Table 9). All 12 MYB genes were differentially expressed at the transcript level but were obviously upregulated in the russet fruit skin of sand pear. Additionally, our study detected the differential expression of three WIN1/SHN1 homologs that belong to the AP2/EREBP family, which are putative direct regulators of cutin and wax biosynthesis^60^. As their transcripts were all repressed in russet fruit skin (Supplemental Table 9), these WIN1/SHN1 homologs may contribute to the periderm suberization process in russet skin. In summary, taking green fruit skin (with cuticle covering) as a reference, two types of metabolic and signaling pathway changes occurred in the russet fruit skin of sand pear: signals that modulate changes in metabolic signaling pathways and stress responses, including programmed cell death. The mutant gene that induced the trait variation between the russet and green fruit skins was regarded as the node that links the two types of signals. Mapping studies of pear fruit skin russet identified two QTLs in LG16 (refs. ^61^) and LG8 (refs. ^62,63^), which supplies the potential genetic locus for the trait mutation. A set of genes have been screened as russet candidates, including the KCS gene localized in LG8 (refs. ^6–8^). A major QTL for apple fruit skin russet has been mapped on LG12^64^, and a putative ABCG family transporter was identified nearby, which is not collinear with the sand pear LG8. The russet trait could be controlled by different genes in the homologous species^61–64^. Further fine mapping of the russet QTLs in pear is needed for candidate gene identification.

## Conclusions

The CM and PM are crucial due to their polyester barrier properties that allow terrestrial plants to adapt to various environmental stresses. The mutations between CM and PM in pear fruit skin make it a suitable material for uncovering and comparing the regulatory mechanisms underlying PM and CM formation. Combined omics analysis yielded a two-dimensional expression profile at both the transcript and protein levels for the two types of fruit skins, and candidate genes and pathways involved in the biosynthesis and regulation of the components of suberin and cutin, and complex stress tolerance networks. The enriched homologs provided clues of new biological roles for well-characterized genes. Both biotic and abiotic stress tolerance genes underlying the russet fruit skin of sand pear were put forward, supplying candidates for further manipulation of both signals and biochemical substances of certain tissues and organs in an attempt to develop plants that are more tolerant to stresses. The study of the proteome of sand pear fruit skin identified new genes and regulatory mechanisms of certain metabolic signaling pathways and stress tolerance under trait mutations, filling the gaps of previous studies. The combined omics analysis of the CM and PM also represents a valuable source of knowledge, regarding plant environmental adaptation from primary to secondary development and cork biology.

## Supplementary information

Supplementary Fig. S 1

Supplementary Fig. S 2

Supplementary Fig. S 3

Supplementary Fig. S 4

Supplementary Fig. S 5

Supplementary Table S1

Supplementary Table S2

Supplementary Table S3

Supplementary Table S4

Supplementary Table S5

Supplementary Table S6

Supplementary Table S7

Supplementary Table S8

Supplementary Table S9
