# Supplementary material for: Proteome and transcriptome profile analysis reveals regulatory and stress-responsive networks in the russet fruit skin of sand pear
Source: Hortic Res. 2020 Feb 1;7:16. doi: 10.1038/s41438-020-0242-3 (PMC6994700; doi:10.1038/s41438-020-0242-3)
Supplement: Supplementary file 2 — Supplementary Fig. S 2 [file 41438_2020_242_MOESM2_ESM.pdf]

Regulatory and environmental adaptation mechanisms in the russet fruit skin of sand pear

Yuezhi Wang*, Meisong Dai, Danying Cai, Zebin Shi*

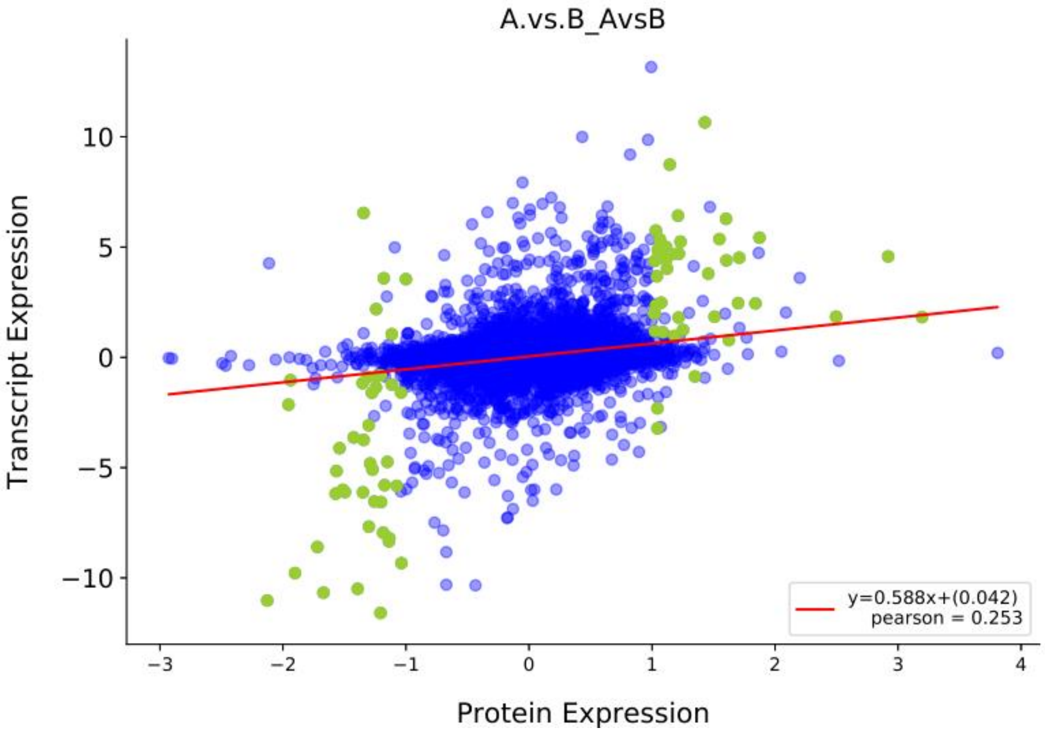

Supplementary Fig. S2 Concordant tests revealed a relative low correlation between 10,881 pairs of proteins and cognate mRNAs.
