# Supplementary material for: Proteome and transcriptome profile analysis reveals regulatory and stress-responsive networks in the russet fruit skin of sand pear
Source: Hortic Res. 2020 Feb 1;7:16. doi: 10.1038/s41438-020-0242-3 (PMC6994700; doi:10.1038/s41438-020-0242-3)
Supplement: Supplementary file 3 — Supplementary Fig. S 3 [file 41438_2020_242_MOESM3_ESM.pdf]

Yuezhi Wang*, Meisong Dai, Danying Cai, Zebin Shi*

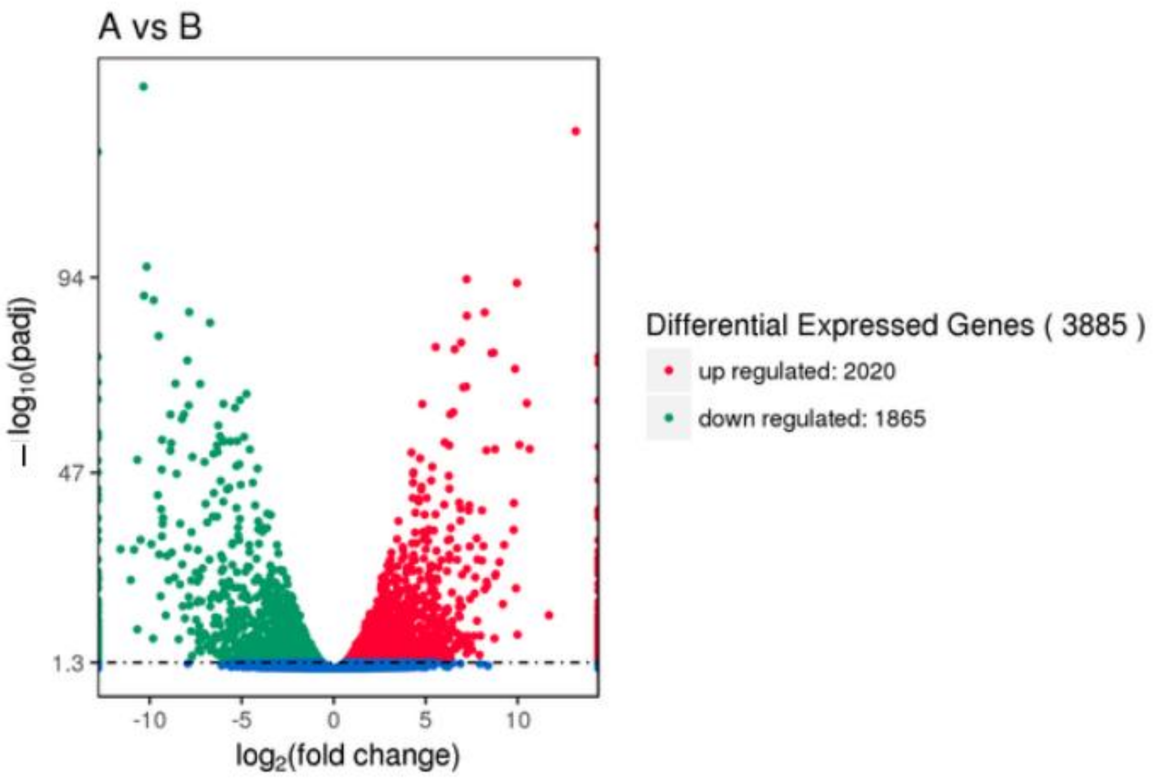

L

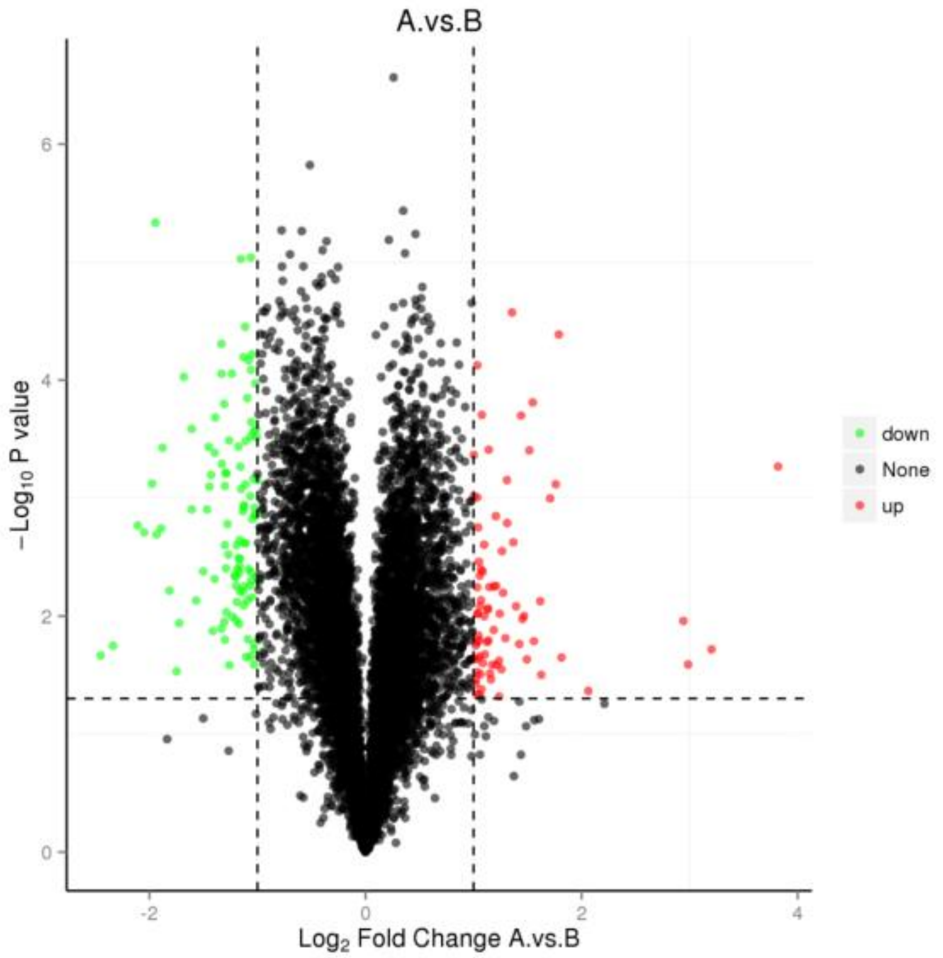

R

Supplementary Fig. S3 Spot distribution of different expression genes at transcript (L) and protein (R) level. The significantly differentially expressed genes are indicated by red (up-regulated) and green (down-regulated) dots, genes with no significant difference are shown in blue or black dots. The abscissa represents the multiple changes of gene expression between the russet (A) and the green (B) fruit skin samples, the ordinate represents the statistical significance of the variation of gene expression.
