# Supplementary material for: Proteome and transcriptome profile analysis reveals regulatory and stress-responsive networks in the russet fruit skin of sand pear
Source: Hortic Res. 2020 Feb 1;7:16. doi: 10.1038/s41438-020-0242-3 (PMC6994700; doi:10.1038/s41438-020-0242-3)
Supplement: Supplementary file 4 — Supplementary Fig. S 4 [file 41438_2020_242_MOESM4_ESM.pdf]

Regulatory and environmental adaptation mechanisms in the russet fruit skin of sand pear

Yuezhi Wang*, Meisong Dai, Danying Cai, Zebin Shi*

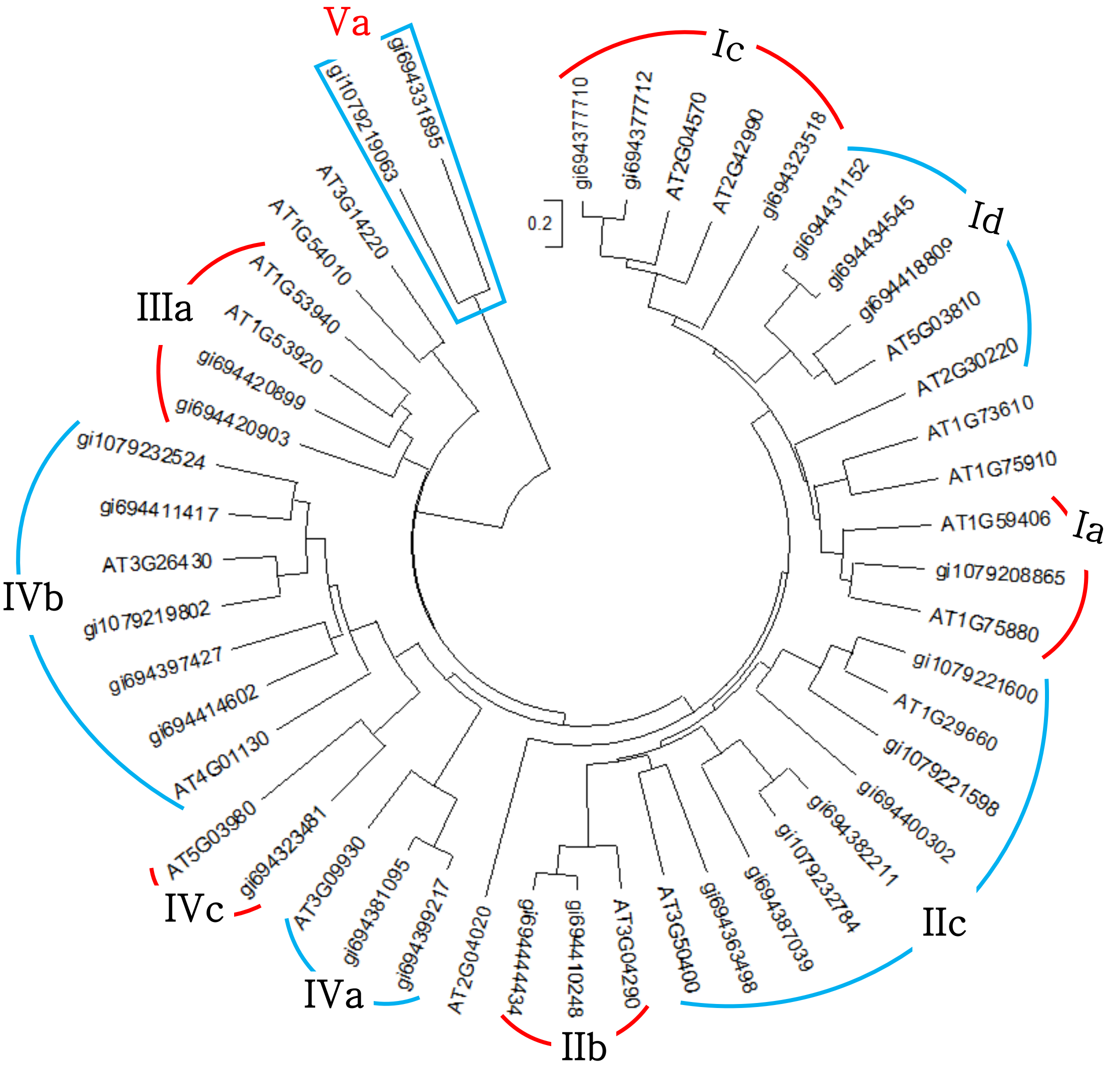

Supplementary Fig. S4 An unrooted tree was constructed based on multiple sequence alignment using the 28 pear GDSL esterase/lipase protein sequences and 19 AtGELPs (Lai et al., 2017) by ClustalW of the Molecular Evolutionary Genetics Analysis (MEGA4) program by NJ method with 1,000 bootstrap replicates. The different types of Arobidopsis GELPs (Lai et al., 2017) were used as the reference in the phylogenetic analysis. Subclades are numbered at crown of the tree and marked with alternating color line to facilitate subclade identification. The subclade Va marked in red was a new subclade firstly identified in this study.
