# Supplementary material for: Proteome and transcriptome profile analysis reveals regulatory and stress-responsive networks in the russet fruit skin of sand pear
Source: Hortic Res. 2020 Feb 1;7:16. doi: 10.1038/s41438-020-0242-3 (PMC6994700; doi:10.1038/s41438-020-0242-3)
Supplement: Supplementary file 5 — Supplementary Fig. S 5 [file 41438_2020_242_MOESM5_ESM.pdf]

Regulatory and environmental adaptation mechanisms in the russet fruit skin of sand pear

Yuezhi Wang*, Meisong Dai, Danying Cai, Zebin Shi*

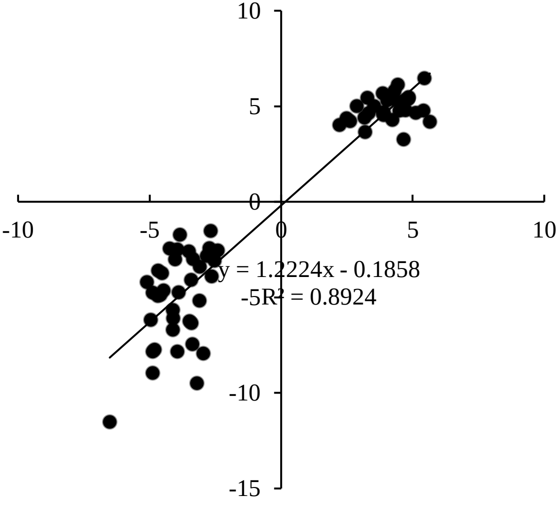

Supplementary Fig. S5 Correlation of gene expression fold change between the russet and green fruit skins analyzed by this study (x axis) with data by Wang et al. (2014a) (y axis).
